# Periodontal disease as a risk factor for sporadic colorectal cancer: results from COLDENT study

**DOI:** 10.1007/s10552-021-01541-y

**Published:** 2022-01-26

**Authors:** Amal Idrissi Janati, Igor Karp, Jean-François Latulippe, Patrick Charlebois, Elham Emami

**Affiliations:** 1 Faculty of Dentistry, Université de Montréal, Montreal, QC Canada; 2 Department of Epidemiology and Biostatistics, Schulich School of Medicine and Dentistry, University of Western Ontario, London, ON Canada; 3 Department of Surgery, Hospital Maisonneuve-Rosemont, Université de Montréal, Montreal, QC Canada; 4 Department of Surgery, McGill University Health Centre, Montreal, QC Canada; 5 Faculty of Dental Medicine and Oral Health Sciences, McGill University, 2001 McGill College Avenue, Suite 500, Montreal, QC H3A 1G1 Canada

**Keywords:** Periodontal disease, Periodontitis, Gingivitis, Colorectal cancer

## Abstract

Colorectal cancer remains the top leading cancer worldwide. Accumulating evidence suggests periodontal pathogens are involved in colorectal carcinogenesis, indicating the need for high-quality epidemiological evidence linking periodontal disease (PD) and colorectal cancer (CRC). Thus, we conducted the first population-based case–control study that was specifically designed to investigate the association between compromised oral health and sporadic CRC. A total of 348 incident cases of colon or rectal cancer, and 310 age and sex frequency-matched controls, from the Montreal island and Laval population participated in the study. Data were collected on PD and on several CRC risk factors using validated questionnaires. A life-course approach was used to document long-term history regarding lifestyle factors. Multivariable unconditional logistic regression analysis was used to estimate the rate ratio (RR) quantifying the association between CRC and PD. Results showed that the rate of new diagnosis of CRC in persons with a positive history of PD was 1.45 times higher than in those with a negative history of PD adjusting for age, sex, BMI, education, income, diabetes, family history of CRC, regular use of non-steroidal anti-inflammatory drugs, lifetime cumulative smoking, lifetime consumption of red meats, processed meats, and alcoholic drinks, and lifetime total physical activity score (adjusted RR = 1.45; 95% CI 1.04–2.01; *p* = 0.026). Our results support the hypothesis of an association between PD and sporadic CRC risk.

**Supplementary Information:**

The online version contains supplementary material available at 10.1007/s10552-021-01541-y.

## Background

Colorectal cancer (CRC) is the third most commonly diagnosed cancer and the second cause of death from cancer worldwide, with over 1,900,000 new cases and over 900,000 deaths in 2020 (Global Cancer Observatory-World Health Organization) [1]. While CRC can develop in inflammatory bowel diseases or hereditary syndromes, most CRC cases are sporadic [2]. Many factors are associated with sporadic CRC, including higher age, male sex, low socioeconomic status, diabetes mellitus, high adiposity, history of CRC in first-degree relatives, tobacco smoking, high consumption of red and processed meat, and heavy intake of alcohol [3–9]. On the other hand, physical activity and use of non-steroidal anti-inflammatory drugs (NSAIDs) decrease the risk of CRC [10–12].

In recent years, studies have suggested that periodontal disease (PD) may increase the risk of CRC. PD is a chronic inflammatory disease, caused by dysbiosis in plaque biofilms, and characterized by progressive destruction of the tooth-supporting tissues [13]. PDs include gingivitis, which refers to gingival inflammation caused by bacteria accumulating in the gingival margin, and periodontitis, where the breakdown of teeth-supporting tissues occurs [14]. The disease manifests in gingival bleeding, clinical attachment loss and radiographically assessed alveolar bone loss, and presence of periodontal pockets. Severity and extent of clinical manifestations increase with disease progression and can reach a high level of tooth mobility and, ultimately, tooth loss in advanced stages [13]. Beyond oral health, PD seems to also impact some extra-oral health outcomes and has been found to be associated with major systemic diseases, including cardiovascular, respiratory, chronic kidney, and metabolic diseases, adverse pregnancy outcomes, rheumatoid arthritis, and cancers [15]. The putative mechanism of PD and cancer association involves the spread of periodontal pathogens to extra-oral sites, dissemination of bacteria endotoxins, and release of inflammation products directly into the bloodstream. Chronic inflammation, on the other hand, promotes carcinogenesis by induction of gene mutations, inhibition of apoptosis, stimulation of angiogenesis, cell proliferation, and epigenetic alterations [16, 17]. PD has been linked to gastrointestinal cancers, but the strength of evidence differs across cancer sites [18]. To date, several papers have been published on the association of CRC and PD [19–27], all of which relied on secondary analysis of data from studies that had been designed for other purposes. These papers suffer from various methodological limitations including, notably, misclassification of PD status and other relevant characteristics, residual confounding [28], and the low number of documented CRC cases. As a result, the etiologic role of PD in the occurrence of CRC remains uncertain. Thus, to better assess the possible etiologic role of PD in the occurrence of CRC, valid and reproducible epidemiological evidence is needed.

## Objective

The objective of this study was to investigate whether PD increases the risk of sporadic CRC.

## Methods

### Study design

COLDENT study is a “population-based” case–control study that was carried out in the Montreal metropolitan area (Montreal island and Laval), Quebec, from January 2013 to December 2019. The CRC cases were instances of histologically confirmed colon or rectal cancer diagnosed in the 6 months preceding their identification. CRC case identification relied on the assistance of medical staff in surgery and oncology departments of five main hospitals providing CRC care to residents of Montreal island and Laval. The control series was selected by random sampling of age- and sex-based strata of the population of Montreal island and Laval by relying on the Quebec Electoral Office lists during 2013–2019. Specifically, a control-to-case ratio of approximately 1:1 was aimed at across the strata defined by age (within 10 year categories) and sex. The identified/selected subjects were sent the study introductory letter and received a phone call from research staff in the following week. Several callbacks were made for non-responding numbers. The inclusion criteria for both cases and controls were as follows: (1) aged 40–80 years old; (2) resident of montreal island or laval; (3) Canadian citizen; (4) speak English and/or French; (5) no prior diagnosis of cancer; and (6) no prior diagnosis of an inflammatory or a hereditary bowel disease, including lynch syndrome, hereditary non-polyposis colorectal cancer, familial adenomatous polyposis, and related polyposis*.* Eligible respondents who agreed to participate in the study were invited to complete a multi-item study questionnaire in either a face-to-face or phone interview. If an eligible responder was unable to attend the interview, the questionnaire was offered for self-administration. In that case, the participant was instructed on questionnaire completion and was called back by research staff upon receipt of the completed questionnaire. Face-to-face interviews were carried out in research units, at the participant’s home, or in hospitals for CRC patients.

The study was approved by the Research Ethics Committees of all participating institutions, and all study participants were given all the information needed before they signed the study consent form.

### Data collection

Data on PD were collected using eight validated questions that address three categories of information on the subject’s periodontal condition: history of diagnosis or treatment of PD; symptoms or complications of PD; and self-awareness of PD [29, 30] (see Table 1). For each category of information, questions with the highest diagnostic performance indicators were selected, based on results of a previous systematic review by Blicher [30] on validity of self-reported periodontal disease measurements. In a more recent systematic review with meta-analysis, by Abbood [29], on self-reported PD, the estimated pooled diagnostic odds ratios (95% CI) of moderate PD by the questions on previous treatment of PD (deep cleaning); tooth mobility without injury; gum bleeding; and self-awareness of having gum disease were 2.38 (1.35–4.2), 6.99 (3.17–15.43), 1.40 (0.91–2.16), and 3.20 (2.23–4.57), respectively, and 11.72 (4.12–33.36), 2.24 (1.05–4.80), 1.95 (1.25–3.03), and 3.35 (2.17–5.18), respectively, for severe PD [29]. Moderate and severe PD were defined based on the gold standard PD case definition by the Centers for Disease Control and Prevention and the American Academy of Periodontology (CDC-AAP) [31].

**Table 1:**
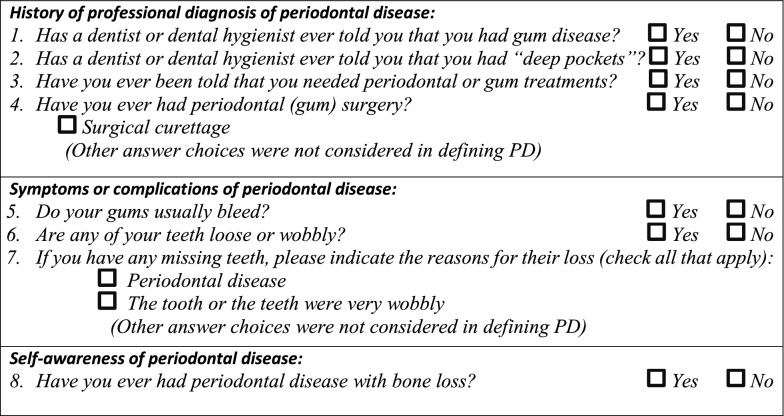
Questions on self-reported periodontal disease

Further, seven study-questionnaires were administered: Sociodemographic and Medical History, Smoking, Height and Weight, Anti-inflammatory Medications, Oral Health, Food Frequency (FFQ), and Lifetime Total Physical Activity (LTPAQ) questionnaires [32–36]. Through administration of these questionnaires, we collected data on history of cigarette smoking, including age started, age ended, years quit during period of usage, and intensity (number of cigarettes smoked per day, per week, or per month). Participants were asked to report all occupational, household, and recreational activities they had done in their lifetime. The minimum threshold for an activity to be reported in the LTPAQ is 124 h/year for occupational, 112 h/year for household, and 32 weeks/year for recreational activities [37]. Each activity was described in terms of duration (age started and age ended), frequency (number of hours per week, weeks per month, and months per year of activity practice), and intensity: light, moderate, and vigorous. Weak intensity was only used for occupational activities to describe those that require sitting with minimal walking.

For dietary risk factors, data were collected on intake of different kinds of red meats and processed meats and of different alcoholic beverages since adulthood. Specifically, red meats referred to meat in hamburgers, beef, pork, and lamb; processed meats referred to bacon, hot dogs, or other kinds of processed meats as salami, bologna, and sausages; and alcoholic drinks referred to beer, wine, and liquor. The FFQ was then administered for four age periods: 20–34, 35–49, 50–64, and 65–80 years. Interviewers relied on the lifetime grid technique to enhance recall accuracy [38].

### Coding of data on periodontal disease and covariates

Subjects were classified as having a positive history of PD if they reported previous professional diagnosis or treatment of PD, and/or if they had experienced either frequent gingival bleeding, or tooth loss caused by tooth mobility or PD, and/or if they were self-aware of having PD. Periodontal health status of participants who answered Yes to only question 6 about tooth mobility (see questions in Table 1) was considered as unknown since tooth mobility could also be caused by an occlusal trauma in a healthy periodontium. Participants were considered as “unexposed” to PD if none of the responses they provided to the 8 questions was positive.

Covariates for adjustment included age, gender, education attainment (elementary school vs. high school and ≥ college), annual personal income, body mass index (BMI), history of type II diabetes, history of CRC in first-degree relatives, history of regular use of NSAIDs (Yes/No), lifetime measure of cigarette smoking (as quantified by packs-years), lifetime measures of consumption of red meats, processed meats, and alcohol, as well as lifetime cumulative physical activity score.

Regular use of NSAIDs was defined as use of at least one tablet/capsule of NSAIDs per month for six continuous months or longer, of aspirin and non-aspirin NSAIDs (NA-NSAIDs). The number of packs-years was calculated as the product of the number of cigarettes smoked per day divided by 20 and the number of years smoked. Lifetime measures of consumption of red meats, processed meats, and alcoholic drinks were calculated as the average number of consumed weekly servings for red and processed meats, and of daily drinks for alcohol, since the participant was 20 years old.

Lifetime physical activity score was represented by the average amount of total energy expended during occupational, recreational, and household activities, expressed by metabolic-equivalent of task (MET) in MET-hour/week/year [37, 39–41]. For this, we assigned MET values to the intensity of physical activities: weak as 1.5, light as 2.5, moderate as 4, and vigorous as 8. To consider both the effects of duration and intensity, each activity was converted into energy expended by multiplying its assigned MET value with the reported hours spent in the activity per year, and the number of years the activity lasted. All subject activities were then summed to derive subject lifetime cumulative energy expended in MET-hours-years. This cumulative measure was then divided by the individual’s age (in years) and by 52 (i.e., the number of weeks in a year) to derive physical activity scores in MET-hour/week/year.

### Statistical analysis

The distributions of potential confounders in the case and control series were examined by calculating the median and inter-quartile range for continuous variables, and percentage for categorical variables.

We fitted multivariable unconditional logistic regression models to estimate the rate ratio (RR) quantifying the association between CRC and PD. Specifically, in the multivariable models, the RR was adjusted for the matching variables (age and sex) and for all the other potential confounders, namely, education attainment, annual personal income, BMI, history of type II diabetes, history of CRC in first-degree relatives, history of regular use of aspirin and NA-NSAIDs, lifetime measure of cigarette smoking, lifetime measure of consumption of red meats, lifetime measure of consumption of processed meats, lifetime measure of consumption of alcohol, and lifetime cumulative physical activity score. The linearity in the logit was assessed for all continuous independent variables, namely age, BMI, annual personal income, lifetime measure of consumption of red meats, processed meats, and alcoholic drinks, and lifetime physical activity score, using the Box-Tidwell test, which involves adding simultaneously interaction terms of each continuous variable and its natural logarithm (Xi multiplied by ln(Xi)) to the multivariable regression model [42]. The test revealed that none of the interaction terms involving the above-mentioned continuous covariates was statistically significant at the alpha level of 0.05.

The percentages for missing data for each variable were less than 10%, except for regular use of aspirin (23%), which was absent from the NSAID questionnaire during the first year of the study (see Supplementary Table S1). Missing data were addressed with the multiple imputation method using the Expectation–Maximization with Bootstrapping algorithm [43]: 10 complete datasets were generated to produce pooled “final” RR estimates along with their corresponding 95% confidence intervals (CIs). To improve performance of the imputation algorithm, auxiliary variables were included for imputation, in addition to all the variables included in the “associational” models. Continuous variables with asymmetric distribution, or extreme values, as for cumulative cigarette smoking, lifetime measures of consumption of red meats, processed meats, and alcoholic drinks, were log-transformed (Log10 (Xi) or Log10 (Xi + 1)) before imputation. Imputed values of continuous variables were restricted to the observed minimum and maximum values. Imputation was done respecting the scale of each variable (i.e., continuous, ordinal, or categorical) with the Amelia II package in R, version 3.5.3 [44].

## Results

**Fig. 1:**
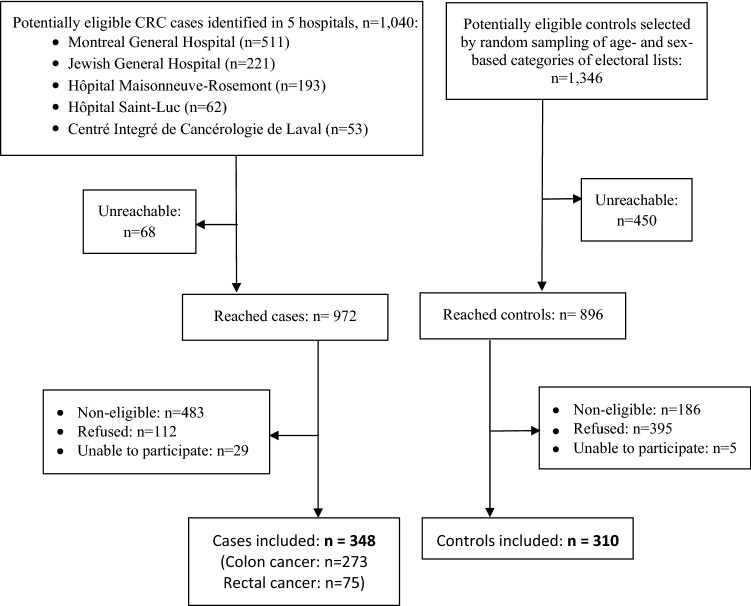
Flow chart of COLDENT study recruitment

A total of 1040 potentially eligible cases of CRC were identified from the five participating hospitals. We approached 972 patients, of whom 483 did not meet the study eligibility criteria, mainly because of their residence area (*n* = 109), previous diagnosis of cancer (*n* = 105), long time since CRC diagnosis (*n* = 93), age (*n* = 89), and language criteria (*n* = 56). Twenty-nine were not able to participate (16 were too ill or too busy because of their cancer treatments, eight had a cognitive or mental illness, five died between the time of their identification and the time when we attempted to reach them or meet for an interview), and 112 refused to participate. Thus, 348 CRC patients took part in the study. During the same period, 1346 CRC-free controls were sent the introduction letter. We were unable to reach 450 subjects, including 119 subjects whose contact details (address and/or phone number) had changed, and five subjects who had died between the time of their selection and the time when we attempted to reach them. Among 896 subjects who were reached, 186 did not meet the study eligibility criteria (mostly because of a previous cancer (*n* = 79), and not speaking French or English (*n* = 52)), five subjects were unable to participate because of a cognitive condition, and 395 refused to participate. Thus, 310 control subjects took part in the study (see flow chart in Fig. 1).

Table 2 presents the distributions of the CRC cases and controls (both overall and according to their PD-history status) according to sociodemographic and other relevant characteristics. Regarding sociodemographic and anthropometric characteristics, cases and controls showed similar mean age (63 years old) and BMI (26 kg/m^2^), and similar relative frequencies of lifestyle and family history of CRC in first-degree relatives. However, the case series showed higher relative frequencies of male gender, elementary and high school education, and history of type II diabetes. Further, the median personal income in the cases was lower than in the controls. The relative frequency of regular use of aspirin was higher in the cases than in the controls, while the relative frequency of regular use of NA-NSAIDs was lower in the cases than in the controls. Considering assessed behavioral CRC risk factors in lifetime, the cases showed a higher proportion of smokers, higher average number of packs-years, higher level of intake of processed meats, and higher average MET-hour/week/year scores in their occupational activities.

**Table 2:** Sociodemographic characteristics and CRC risk factors in COLDENT study participants

Characteristic	Cases, *n* = 348 *n* (%)	Controls, *n* = 310 *n* (%)		
	Total	Total	Positive history of PD *(n* = 117*)*	Negative history of PD *(n* = 183*)*
Age, years (mean (SD))	63.2 (9.8)	63.1 **(**9.8)	61.4 (9.6)	63.7 (9.8)
Gender				
Male	215 (62)	170 (55)	66 (56)	98 (54)
Canadian born				
Yes	216 (62)	210 (68)	77 (66)	126 (69)
Native tongue				
French	155 (46)	183 (59)	63 (55)	112 (64)
English	60 (18)	40 (13)	20 (17)	20 (12)
Other language	123 (36)	75 (24)	32 (28)	42 (24)
Education attainment				
Elementary school	29 (9)	9 (3)	4 (3)	5 (3)
High school	116 (34)	92 (30)	36 (31)	53 (30)
College or University	197 (58)	199 (64)	76 (65)	117 (67)
Living alone				
Yes	71 (21)	80 (26)	27 (23)	47 (26)
BMI, kg ∕m^2^ (mean (SD))	26.1 (5.7)	26.3 (6.2)	27 (5.7)	27.4 (4.7)
Family history of CRC				
Positive	43 (12)	38 (12)	13 (11)	24 (13)
History of diabetes				
Positive	69 (20)	39 (13)	14 (12)	23 (13)
History of regular use of NA-NSAIDs				
Positive	62 (18)	67 (22)	28 (25)	39 (21)
History of regular use of aspirin				
Positive	85 (27)	50 (26)	16 (21)	33 (30)
Personal income (CAD$ per year)	35 000 (50 000)	45 000 (40 000)	45 000 (50 000)	45 000 (40 000)
History of cigarette smoking				
Positive	206 (59)	174 (56)	73 (62)	97 (53)
Cigarette smoking, packs-years (Median (IQR))	3.6 (24)	2 (22)	2.2 (17.5)	1.8 (23.5)
Lifetime average daily alcoholic drinks^a^ (Median (IQR))	0.4 (1.1)	0.5 (1)	0.4 (0.9)	0.5 (1)
Lifetime average weekly servings^b^ of:				
Red meats (Median (IQR))	4.3 (3.5)	4 (3.5)	4.4 (3.5)	3.9 (3.3)
Processed meats (Median (IQR))	2.6 (3.5)	1.8 (3.3)	1.8 (3.2)	1.8 (3.3)
Lifetime average MET hour/week/year (Median (IQR))				
Occupational	60.3 (51.6)	48.7 (40.8)	50.5 (41.5)	46.9 (36.5)
Household	13.4 (27.6)	13.1 (25.1)	12.3 (26)	13.6 (23.7)
Recreational	8 (14)	7.7 (12.8)	8.4 (12.7)	6.5 (11.3)
Total physical activity	95.7 (78.2)	85 (53)	84.8 (44.9)	84.9 (53.6)

The frequencies presented in the table correspond to valid percentages

*CRC* colorectal cancer, *BMI* body mass index, *NA-NSAIDs* non-aspirin non-steroidal anti-inflammatory drugs, *CAD$* Canadian dollar, *MET* metabolic equivalent of task, *SD* standard deviation, *IQR* interquartile range

^a^One drink include beer (355 ml bottle or can), wine (180 ml), or liquor (150 ml)

^b^One serving of red meats = 180–240 g, one serving of processed meats = 55 g

The prevalence of PD, including gingivitis and periodontitis, was higher in the case group (46%) than in the control group (38%). Overall, cases were more likely to report previous diagnosis or a symptom of PD, or to be aware of having PD with bone loss, than controls. The complication of advanced PD, tooth loss due to mobility or PD, was less frequent (8% in cases and 4.8% in controls).

Results from multiple logistic regression analysis showed that the rate of (new diagnosis of) CRC in persons with a positive history of PD was 1.4 times higher than in those with a negative history of PD when adjusting only for age and gender (adjusted rate ratio (RR_a_ = 1.4; 95% CI 1.02–1.91; *p* = 0.037) and 1.45 times higher than in those with a negative history of PD when adjusting, in addition, for BMI, education, income, diabetes, family history of CRC, regular use of aspirin and NA-NSAIDs, lifetime cumulative smoking, lifetime consumption of red meats, processed meats, and alcoholic drinks, and lifetime total physical activity score (RR_a_ = 1.45; 95% CI 1.04–2.01; *p* = 0.026). The results are summarized in Table 3.

**Table 3:** Rate ratio (RR) for association between periodontal disease and colorectal cancer

	RR (95% CI); *p* value
Adjusted for age and sex	1.4 (1.02–1.91); *p* = 0.037
Adjusted for all covariates^a^	1.45 (1.04–2.01); *p* = 0.026

^a^Age, sex, education attainment, annual personal income, BMI, history of type II diabetes, history of CRC in first-degree relatives, history of regular use of aspirin and non-aspirin non-steroidal anti-inflammatory drugs, lifetime measure of cigarette smoking, lifetime measure of consumption of red meats, lifetime measure of consumption of processed meats, lifetime measure of consumption of alcohol, and lifetime cumulative physical activity score

## Discussion

In this case–control study, we investigated the association between PD and CRC, and relied on multiple self-reported measurements to ascertain the PD status. Our findings suggest that the CRC rate is increased in persons with a positive history of PD compared with persons with a negative history of PD, even upon adjustment for a number of potential confounders. Studies that previously examined the association between CRC and self-reported PD [22, 23, 25, 26] assessed PD status with only one question, which was about previous professional diagnosis of PD, among a cohort of American male health professionals and a cohort of elderly women [23, 26]; self-awareness of periodontal bone loss in American women nurses [22]; or a clinical sign of PD (tooth mobility with consequent tooth loss) in a cohort of adult Sweden twins [25]. Unlike those studies, we ascertained self-reported PD exposure using a combination of questions that concerned the three types of information together. Using only one question can be expected to result in PD-status misclassification, especially in persons with lower education or socio-economic level [45]. For example, questions related to professional diagnosis will favor accurate answers in subjects who had more access to dental care. [29, 30] Thus, persons who have not visited a dentist in years may not report any previous diagnosis of PD and will be classified as unexposed, while in fact they could well have the disease, especially given their poorer access to, and lower levels of use of, dental care. According to the Canadian Health Measures Survey 2007–2009, 48% of Canadian adults who have not been to a dental professional in last year had gingivitis, and 48% of Canadian adults from the lower income group had gingivitis, compared with 25% of Canadians with higher incomes [46]. Questions about the most easily perceived clinical signs, such as tooth mobility, or tooth loss caused by mobility, and PD have greater informativeness in detecting PD but may result in failure to detect early stages of the disease, including gingivitis and mild periodontitis. This justifies questioning on gingival bleeding as well. Self-awareness of one’s own PD condition is more informative among educated people and dental or health care users. To conclude, combining different questions—as recommended by Blicher et al. [30] and Abbood et al. [29] in their systematic reviews’ conclusions—will enhance the ability of the questions to identify history of PD. Those authors also recommended using variations of the same question in order to stimulate the person’s memory; thus, we used different questions to retrieve a previous diagnosis or treatment of PD.

According to our self-reported PD definition, we found that 38% of controls had either gingivitis or periodontitis, and if we refer to those who reported tooth loss because of mobility and PD, we can consider 5% of controls had advanced PD. These statistics seem to be in line with those from the Canadian Health Measures Survey (2007–2009), where partial‐mouth periodontal examination was used, and which estimated that 32% of Canadian adults (20–79 years of age) had gingivitis, 21% of adults with natural teeth had, or had had, a moderate or a severe periodontal (gum) problem, and 4% of Canadian adults had severe periodontal disease [46]. According to complete-mouth examination in the National Health and Nutrition Examination Surveys (2009–2014), and the CDC-AAP case definition [31], the estimated prevalence of total periodontitis in dentate US adults aged 30–79 years was 42%, including 8% severe periodontitis.

We found a statistically significant association of CRC with PD (RR_a_ = 1.45; 95% CI 1.04–2.01) (*p* = 0.026), adjusting for a number of potential confounders. Similarly, two previous studies have reported a positive association of CRC with PD. The first is a large retrospective cohort study (*n* = 106,487) based on administrative data from Taiwan’s National Health Insurance Research Database [19]. The estimated hazard ratio (HR) (95% CI) was 1.64 (1.50–1.80), adjusting only for age, gender, and comorbidity. PD classification in that study relied on established administrative codes given to clinical diagnosis of gingivitis and periodontitis, and no additional information was provided on any standardized clinical measures or definition of PD. In addition to this, and to the high risk of residual confounding due poor adjustment, using the Insurance Health database was criticized in another report for the significant risk of misclassification due to deliberate over-coding by health and dental care providers to avoid refusal of reimbursement by medical insurance [47]. The results of this study should thus be interpreted with great caution. In the second study, Arora [25] analyzed data from a large Swedish prospective cohort study of homo- and heterozygous twins, which had been originally designed to study the role of environmental and genetic factors in cardiovascular disease and cancer. Their analysis included 15,333 twins who answered the question on periodontal status: “Have you noticed that some of your own teeth have come loose or fallen out on their own?” Participants were then classified as having PD if at least half of their teeth were wobbly, indicating that PD is at an advanced stage. Participants who reported having a few loose teeth were separately classified as having minor mobility. Almost 6% of participants had advanced PD and an additional 12% of participants reported minor tooth mobility. Participants had a median age at study entry of 51 years and followed-up for a median period of 27 years, with a total of 200 CRC cases documented. Upon adjustment for gender, age, education, employment, number of siblings, smoking status (5 categories counting number of packs per day), smoking status of partner, alcohol status (current, former, never), body mass index (4 categories), and diabetes, the estimated HR (95% CI) was 1.62 (1.13–2.33). Unfortunately, the list of potential confounders adjusted for did not include dietary risk factors and physical activity level. Furthermore, the PD measure in that study excluded gingivitis and early-stage periodontitis.

Different results were reported by Momen-Heravi [22], who analyzed data from the American Nurses’ Health Study. The analysis included a subsample of 69,656 participants who were asked if they had a history of periodontal bone loss (in study cycle of 1998). Where the answer was yes, participants indicated the severity of the bone loss (none, mild, moderate/severe). After 18 years of follow-up, a total of 739 CRC was documented for this analysis. Overall, the study results suggested no association between history of periodontal bone loss and CRC (HR = 0.89; 95% CI 0.72–1.10), although they were weakly suggestive of positive association for moderate/severe bone loss and CRC (HR = 1.22; 95% CI 0.91–1.63), adjusting for age, ethnicity, smoking, history of CRC in first-degree relatives, history of sigmoidoscopy/colonoscopy, current physical activity, regular aspirin use, multivitamin use, diabetes, alcohol consumption, BMI, energy-adjusted intake of total calcium, vitamin D, folate, red meat and processed meat, and hormonal replacement therapy). The study indeed has many strengths, such as its prospective design, large sample size, and adjustment for numerous potential confounders.

Results from these epidemiological studies on the association between PD and CRC (or colorectal adenoma [48, 49]) have been synthesized in a recent meta-analysis, by Xuan [50]. Specifically, these authors reported that periodontal disease was statistically significantly associated with colorectal tumor (pooled “relative risk” and 95% CI: 1.25 (1.06–1.38), although there was high heterogeneity across studies (*I*_2_ = 83.9%) [50].

The oral cavity can indeed serve as a reservoir for the systemic dissemination of pathogenic bacteria and their toxins, leading to infections and inflammations in distant bodily sites, and several oral species were identified in infections at extra-oral sites [51]. Scannapieco and Panagakos [52] suggested four potential pathways that may allow oral bacteria and gingival inflammation to influence systemic health, including bacteremia, systemic spread of locally produced inflammatory mediators, eliciting an autoimmune response, and aspiration or ingestion of oral bacteria into the intestine or respiratory tract [52].As for CRC, a periodontal pathogen, *Fusobacterium nucleatum*, has been particularly involved in CRC tumorigenesis, and its presence in colorectal mucosa and feces has been found to be associated with CRC [53]. The development of CRC is, on the other hand, strongly influenced by the inflammatory condition of the colon, as shown in patients with inflammatory bowel disease, where chronic and severe inflammation of the colon increases their risk of developing CRC [54].

To our knowledge, the COLDENT project was the first epidemiological study specifically designed to assess the association between PD and CRC. In the study we used rigorous methods for documentation of the main exposure, study outcome, and the potential confounding factors. Moreover, we followed a life-course approach to document long-term history regarding lifestyle factors and other relevant characteristics. We also chose to assess PD based on self-reported measures, because they allow easier standardization of the exposure measurements and definition than dental examination, which is sensitive to inter- and intra-examiner variation.

Our results support the hypothesis of an association between PD and sporadic CRC risk. Further epidemiological studies aimed at production of high-quality evidence on the putatively causal relation between PD and CRC occurrence and on the possible mechanisms underlying that relation are recommended.

## Supplementary Information

Below is the link to the electronic supplementary material.

Supplementary file1 (DOCX 12 kb)

## Data Availability

Data supporting the findings of this study will be available if the institutional review boards accept and upon a request from the corresponding author.

## Abbreviations

BMI: Body mass index
CDC-AAP: Centers for Disease Control and Prevention and the American Academy of Periodontology
CI: Confidence interval
CRC: Colorectal cancer
FFQ: Food Frequency Questionnaire
LTPAQ: Lifetime Total Physical Activity Questionnaire
MET: Metabolic-equivalent of task
NA-NSAIDs: Non-aspirin non-steroidal anti-inflammatory drugs
NSAIDs: Non-steroidal anti-inflammatory drugs
PD: Periodontal disease
RR: Rate ratio
RRa: Adjusted rate ratio
