# Supplementary material for: Periodontal disease as a risk factor for sporadic colorectal cancer: results from COLDENT study
Source: Cancer Causes Control. 2022 Jan 26;33(3):463–72. doi: 10.1007/s10552-021-01541-y (PMC8821510; doi:10.1007/s10552-021-01541-y)
Supplement: Supplementary file 1 — Supplementary file1 (DOCX 12 kb) [file 10552_2021_1541_MOESM1_ESM.docx]

| **Table S1. Frequency of missing data in variables included in the regression analysis** | | | |
| --- | --- | --- | --- |
| **Variable** | **Participants with missing data** | | |
|  | **Total, n=658** | **Cases, n=348** | **Controls, n=310** |
|  | **n (%)** | **n (%)** | **n (%)** |
| Periodontal disease | 20 (3) | 10 (3) | 10 (3) |
| Education attainment | 16 (2) | 6 (2) | 10 (3) |
| Personal income | 63 (10) | 36 (10) | 27 (9) |
| BMI | 1 (0.2) | 1 (0.3) | - |
| Regular use of NA-NSAIDs | 6 (1) | 1 (0.3) | 5 (2) |
| Regular use of aspirin | 154 (23) | 37 (11) | 117 (38) |
| Cigarette smoking | 2 (0.3) | 1 (0.3) | 1 (0.3) |
| Average weekly red meats intake | 34 (5) | 28 (8) | 6 (2) |
| Average processed meats intake | 38 (6) | 30 (9) | 8 (3) |
| Average daily total alcoholic drinks | 32 (5) | 27 (8) | 5 (2) |
